# “Meeting a human, not a medical condition”: a qualitative study of simulated patients’ perceptions of forming a therapeutic alliance with psychiatrists

**DOI:** 10.1186/s41077-026-00446-7

**Published:** 2026-05-23

**Authors:** Sydney Potts, Mona Gupta, Nadia Daly

**Affiliations:** 1 University of Toronto, Toronto, Ontario Canada; 2 Université de Montréal, Montréal, Québec Canada; 3 Autisme MD, Montréal, Québec Canada

**Keywords:** Simulated patients, Psychiatry, Therapeutic alliance, Education, Training, Psychotherapy, Bond

## Abstract

**Objective:**

Increasingly in psychiatric and psychotherapeutic training, simulated patients (SPs) participate in the teaching and evaluation of clinical skills and knowledge. Despite their widespread involvement, doubt remains as to whether a genuine therapeutic alliance can be established with SPs. Further, little is known about the SP’s perspective on alliance formation which is an important gap given the correlation between patient perception of alliance and therapeutic success.

**Methods:**

We interviewed three simulated patients about their perception of the bond formed during role plays of standard full-length psychiatric diagnostic assessments. Each SP played their role multiple times yielding sixteen unique interactions and therefore sixteen interviews. SPs watched a complete video recording of each of their role plays. After watching each role play, they rated their interaction with the psychiatrists using the bond subscale of the Working Alliance Inventory (WAI). Following the ratings, each SP was interviewed about the bond formation in each interaction.

**Results:**

Despite the simulation, SPs were able to form bonds with psychiatrists across full-length diagnostic assessments. Feeling respected by the psychiatrist, both in the psychiatrist’s attempt to understand the problem and in their approaches to finding solutions facilitated bond formation. However, SPs had different preferences as to how respect should be conveyed. When it went well and when it went wrong, bond formation was affected by the same factors in the simulations as is the case in real practice.

**Conclusions:**

Our results suggest that from the point of view of SPs, a therapeutic alliance as reflected by bond formation can be established between psychiatrists and SPs in the context of full-length diagnostic assessments. These findings would be strengthened through replication involving comparison of both the SP and the psychiatrist perspectives.

**Supplementary Information:**

The online version contains supplementary material available at 10.1186/s41077-026-00446-7.

## Background

Training with Simulated Patients (SPs) has become an essential component of psychiatric education and medical education more broadly. While SP interactions are considered reasonable proxies for some types of patient encounters, there may be important challenges — particularly with respect to relational authenticity — which raise doubts as to their appropriateness for the teaching and assessment of certain advanced competencies. As a result, the uptake of SPs in postgraduate psychiatry and psychotherapy training has generated ongoing debate.

SP performance-related factors have been a recurring concern for researchers working at the intersection between simulation-based education and psychiatric and psychotherapeutic training [1] including difficulties portraying complex thought disorders, poorly detailed backstories, and overly compliant SPs [2–6]. However, some suggest that relationship-related factors are also relevant, specifically the impact of the simulation on the developing therapeutic alliance (TA) [7]. The TA refers to the relationship formed between the clinician and patient, specifically the emotional bond, and shared tasks and goals [8], and has long been heralded as the foundation for therapeutic success [8]. Trained clinicians and learners reported reduced empathy and emotional engagement in encounters with SPs compared to true patients [9], which could in turn impact the formation of the therapeutic alliance. Interactions with SPs may also teach and reinforce detrimental techniques like performative empathy (expressing understanding without genuine emotional resonance) [3]. Accordingly, teaching and assessing relational capacities such as TA formation with SPs may give inaccurate indications of learners’ abilities [3]. These critiques highlight the impact of relationship-related factors on learner development in SP interactions.

The debate about simulating the TA has focused on the experiences of clinicians, leaving the SP perspective about the quality of the relationship largely overlooked. However, the patient’s perspective of the therapeutic alliance is one of the strongest predictors of its strength [10], and is equally important in diagnostic evaluation, clinical follow-up and psychotherapy [11–13]. Integration of SP interactions in psychiatric and psychotherapeutic training therefore requires consideration of how SPs perceive the quality of the relationships they form with clinicians. Scholars have identified the inclusion of SPs perspectives in simulation research as a critical gap in the literature [14] and is arguably even more essential when assessing relationship-related factors in particular. Given the centrality of the patient’s perspective on the alliance in determining the effectiveness of psychiatric and psychotherapeutic work, SPs accounts of the alliance also represent a vital source of formative feedback for learners.

Some studies have reported on SP’s experiences across various disciplines of medical training [15, 16]. For example, some work suggests SPs may experience negative psychological impacts as a result of their role plays [15]. However, research examining how SPs experience the TA in working with psychiatrists, especially in realistic long interviews, has yet to be undertaken. This study sought to identify the factors that influence the bond component of the TA from the SP’s perspective during a full-length diagnostic assessment with psychiatrists. In relation to the existing literature, this study’s setting more closely approximates the context and duration of real clinical encounters as compared to the usual OSCE setting of SP research.

## Methodology

### Design

We undertook a video-elicited interview-based qualitative study of SP’s perceptions of the factors that impact bonds between themselves and psychiatrists in full-length diagnostic encounters. Our approach aligns with a constructivist framework given that bond formation is intersubjective and the reporting of bonds in research interviews itself depends on the relational experience between participants and interviewers. We analyzed semi-structured interviews with three SPs about the bond they formed with different psychiatrists in the diagnostic reasoning study described below. SP1 and SP3 performed their role with five different psychiatrists, and SP2 performed her role with six psychiatrists for a total of 16 interviews involving 16 different psychiatrists.

### Diagnostic reasoning study (DRS) participants

In the original DRS, sixteen psychiatrists (men *n* = 11) and (women *n* = 5) in a French language tertiary care hospital in Montreal, Canada were recruited to participate in a full length diagnostic assessment of a new patient. They were recruited by an email notice sent by a research assistant to all psychiatrists and psychiatry residents in that institution. They ranged in experience from residents in their second year to senior clinicians. Clinicians from different practice areas of psychiatry were represented including general psychiatry, mood disorders, psychotic disorders, consultation-liaison psychiatry, and geriatric psychiatry.

### Simulation

Three professional actors (hereafter referred to as SPs) were recruited to each play a distinct patient role for the assessment (see Appendix A in Supplementary Information for role descriptions). The SPs’ role descriptions were developed by MG, a psychiatrist and study researcher. After having received their scenarios in advance, the SPs participated in a full day of training with MG and a research assistant. First the group discussed the scenarios. Each SP then practiced their roles in a full-length diagnostic assessment and received feedback from the psychiatrist (MG), the research assistant, and each other about the authenticity of their role portrayal. The actors also gave each other feedback relating to portrayal of the patients’ backstories and modifications were made to their scripts as a result.

The assessments took place over a 12 month period from January 2014 to January 2015. They occurred at the psychiatrists’ offices at a time during clinic hours. The assessments lasted between 45 and 60 min and were video recorded by a research assistant who was in the room during the assessment. The psychiatrists were aware that the patients were SPs, and all participants (clinicians and SPs) were aware that the study was investigating the diagnostic process and reasoning of the clinicians.

Following the simulation, the research team watched the recordings identifying specific points during the interviews that they wished to discuss with the psychiatrists in relation to their diagnostic reasoning. One week after the simulation, each psychiatrist was interviewed individually by the research assistant, regarding their diagnostic process using the ‘retrospective think-aloud’ method [17] whose goal was to elicit their reasoning process during the diagnostic interview. During the research interview, the psychiatrist watched the video along with the research assistant who paused the recording at certain points in order to ask the questions about that moment that had been developed by the team for that specific interview (see Appendix B in Supplementary Information - Interview Guide psychiatrists).

### Current simulated patients study

After each diagnostic assessment in the original DRS, each SP wrote unstructured memos of their experience of the encounter and submitted them to the research team. These notes helped to inform the subsequent psychiatrist-interviews. The team noted that in several memos, the SPs spontaneously commented on the quality of the relationship with the psychiatrist. This gave rise to the question of the present study: What are simulated patients’ perceptions of forming a therapeutic alliance with psychiatrists?

The present study took place between January and April 2015, after all diagnostic assessment interviews from the DRS had been completed. We drew upon a convenience sample of 16 interactions between SPs and psychiatrists that took place during the DRS. SPs watched their video recordings and then completed the bond subscale of the Working Alliance Inventory, (WAI) (see Appendix C in Supplementary Information ).

The WAI is a 36-item, validated measure designed to assess the quality of the therapeutic alliance, typically during an ongoing process of psychotherapy [18]. It is considered to be one of the best measures of the therapeutic alliance [19]. The WAI provides an assessment of the alliance with versions that can be completed from the perspective of the therapist, client, and an observer. For this study we used the client version. The WAI has three subscale measures: agreement on tasks, agreement on goals, and patient-therapist bond [18, 20]. Respondents rate each item on a 7-point Likert scale ranging from 1 (indicating “never” or “rarely true”) to 7 (indicating “always” or “very often true”). However, WAI scores are not standardized meaning a certain score does not indicate a certain strength of the alliance independent of the individual who completes the scale. The only way to evaluate an individual’s ratings is to ask them. Thus, we invited the SPs to participate in semi-structured interviews to explain the rationale behind their strong and weak ratings for each encounter in which they participated (see Appendix D in Supplementary Information - interview guide SPs). In order to mobilize tacit knowledge and contextual details that might be relevant to bond formation, we used the WAI scores and video recordings in combination with the oral questions as an elicitation technique [21].

The SP interviews were transcribed using the digital platform Trint and translated from the original French to English using DeepL. Translations were reviewed and compared to interview recordings by MG, who is English-French bilingual.

### Data analysis

We analyzed the interviews using semantic thematic analysis guided by the approach developed by Boyatzis [22]. Boyatzis’ [22] model applies well when the researcher is interested in semantic (manifest) rather than latent content. Each transcript of the SP interviews was read and reread several times by a first reviewer (SP). Reviewer 1 took notes as to what the SPs said was important in bond formation in each encounter. She then went through each transcript again, identified relational experiences and gave them preliminary codes (e.g., “feeling judged”), staying close to what was explicitly said by the SP. Following this process, she defined each code. A second reviewer (MG) read several transcripts. Both reviewers then discussed each code in order to ensure that each was specific, clearly defined, non-overlapping, and supported across all encounters. Finally, drawing upon these codes, we developed an interpretation of the factors promoting bond formation and the relationships between them, from the SPs’ point of view. We tested this interpretation across each SPs group of encounters to refine it and ensure its applicability. We did not have an external check of trustworthiness but our dual coder process contributed towards the credibility of our analysis.

Because we drew upon a convenience sample, data adequacy was not planned within the study design [23]. Nevertheless, on several dimensions of information power [24] our sample of 16 interviews was sufficient. Specifically, the aim of our study was relatively narrow, the participants’ knowledge was tightly aligned to the aim, and the quality of the dialogue was rich. The fact that the SPs played their own roles several times with different psychiatrists facilitated comparisons of their experiences of bond formation and enabled us to test the robustness of our claims.

### Reflexivity

Our analysis team consisted of one psychiatrist (MG) and one graduate student (SP). The author team shared common perspectives that bond formation is co-created and encounter-specific. We were mindful that our perspectives led us to understand the interactions from a system perspective rather than the result of one party’s behaviour.

## Results

The results section is structured around our main findings about SP’s perceptions of bond formation with the psychiatrists. We provide quotations to illustrate our findings, using anonymous participant codes (P refers to the psychiatrist).

### Respect as foundational to bond formation

Across the interviews, bond formation depended on the SPs feeling respected by the psychiatrist. SPs explicitly located trust as the relational outcome of feeling respected and described trust as necessary to bond formation. As one SP explained, respect was established when the psychiatrist tried to understand the SP’s perspective, rather than foregrounding clinical evaluation:

“He was very much about me and not about medicine or statistics… summarizing things often…There was a lot of listening… In terms of respect, I think that shows respect for the person in front of you…he built trust” (SP2, P6).

By contrast, when the SPs felt disrespected, trust was weakened or even broken. One participant experienced disrespect when confronted about experiencing psychotic symptoms:

“Does he really respect me in this confrontation, in confronting my ideas? Is there really respect, or is there some kind of contempt? …[I]t’s that transition from respect to trust to confrontation. That’s it… the question of trust is at the heart of the interview, and it’s difficult [when I feel confronted]” (SP3, P13).

In these accounts, respect functions as the relational substrate of bond formation. The SPs trusted their psychiatrist when they felt respected, and trust diminished when the SP felt disrespected .

### The many roads to respect

In their interviews, SPs identified the psychiatrists’ empathy, non-judgment, and openness as meaningful insofar as they communicated respect in different ways. These included: treated as credible, taken seriously, feeling accepted, and feeling heard. Thus, respect was not conveyed through a single behaviour or attitude, nor was it necessarily explicit. SPs felt respected through a range of experiences in which respect was sometimes implied.

For instance, one SP describes empathy as valuable because it conveys an acknowledgement from the psychiatrist of the legitimacy of her suffering.

“He was often very empathic, saying things like, ‘If I were you, I would find it difficult too.” (SP2, P11).

Similarly, SP1 received non-judgment as an indication that she was accepted as she was :

“I didn’t feel it was judgmental… I didn’t feel he was judging who I was” (SP1, P2).

Physical and emotional openness contributed to the SP feeling heard.

“I found that P2 and P1 had something in common, which was openness in their posture and physical gaze, which, for me, with my patient and my case, was really essential to feel heard….” (SP1, P1/P2).

Thus, from the SPs’ perspective, empathy, non-judgement, and openness served as communicative vehicles of respect.

### The procedure of respect

For respect to secure a strong bond, it needed to be exemplified within four clinical tasks across two overlapping phases of the diagnostic interview that we named “understanding the problem” and “approaching solutions.”

- Understanding the Problem

SPs thought that psychiatrists communicated respect through two dialogical tasks we called: 1) listening to and understanding the patient’s perspective and 2) acknowledging and believing their emotional experience.

i. Listening to and understanding the patient’s perspective

One participant reflected on the value of listening as a means of conveying respect for her humanity:

“I get the impression that he attaches great importance to you, to why you're there…Which I think is essential because [I] feel like no one believes [me], [I] feel like everyone thinks [I’m] exaggerating, so I think it's important to feel that [the psychiatrist is] meeting a human being and not [a] pathology or a medical condition.” (SP 2, P10)

Conversely, when another SP did not feel understood, the same respect was not conveyed, and the bond failed to form:

“[I felt] like I was standing in front of a cold wall analyzing me…”(SP1, P3)

ii. Acknowledging and believing the SP's emotional experience

During this second task, respect was conveyed by recognizing the SPs credibility on their experience and affirming its significance. At times, this came in the form of affirmation:

“[SP3 quoting P15 as saying] You’re right to be worried. It’s concerning, all of this.” (SP3, P15)

Another SP described the centrality of empathy not only as shared feeling, but as a means of conveying an understanding of and respect for her suffering: “From the outset, he showed a great deal of empathy. He understood the suffering caused by anxiety” (SP2, P11) 

By contrast, when SP1 believed the psychiatrist thought she was exaggerating the significance of her distress, trust was broken and so was the bond:

“[P3 thinks] I'm overplaying my heartbreak. [A]t one point she says, ‘ah, you've got a broken heart, you know, that's basically it, you've got a big broken heart.’ So I don't think she trusts me when I say I need help and all that. I [didn’t] trust her either” (SP1, P3).

b. Approach to finding solutions

During phase two, respect needed to be conveyed through two clinical tasks: 1) collaboration and 2) effective communication

i. Collaboration

It was essential for SPs to believe that the psychiatrists valued and integrated their self-knowledge and preferences when proposing treatment options. 

In one encounter, this manifested in reviewing different treatment options in accordance with the SPs preferences:

“Even when I say I don't like antidepressants, she takes it into consideration…everything I say is really an exchange.” (SP1, P5)

While in another, SP3 reflected on the value and rarity of his provider, centering his care around him:

“Actually, this is the first time, I think, that… I feel that the person in front of me really understands the extent of the problem and the seriousness…and is genuinely concerned with finding a solution that puts me at the center of the solution…” (SP3, P15)

When the SPs felt their perspectives were valued and considered, a bond was formed. However, when that engagement was absent the bond failed. As one SP said,

“It was very one-sided… she would write it down and analyze it… I felt very little feedback” (SP1, P3).

ii. Competence and (In)Effective communication

Finally, bond formation depended on the SPs believing their psychiatrists were equipped to help them, both in knowledge and skill.

For example, the use of medical language to describe SP2’s diabetes signaled competence and inspired confidence:

“[H]is knowledge of diabetes… words like cortisol…to use those terms would automatically inspire confidence. Because there’s a lot of knowledge about the disease” (SP2, P11)

Similarly, when the psychiatrists helped the SPs reflect on their experiences and identify possible strategies, they were perceived as skillful and useful:

“She really helped me a lot to find avenues of reflection and solutions… very skillful.” (SP1, P1)

However, the bond was hindered when the psychiatrist communicated their clinical perspectives in ways the SPs found disrespectful. As one SP recalled,

“[SP2 quoting P7] ‘You’re not easy to reassure.’ I find that a very inhumane way to approach someone who may be suffering from anxiety, to tell them to their face that they are not easy to reassure, because, I was consulting [the psychiatrist precisely because [my] endocrinologist isn't able to reassure [me].” (SP2, P7)

And another reflected:

“When he imposed himself a little in a certain way, I would shut off.” (SP 3, P16)

In these cases, the psychiatrist's attempts to convey knowledge or share their clinical opinions undermined rather than strengthened the relationship. This suggests that, for the SPs, whether psychiatrists’ clinical knowledge facilitated bond formation depended on how it is communicated. 

### Feeling disrespected is person-specific

While each SP felt disrespected at times, the circumstances in which this occurred varied by individual.

For instance, SP1 experiences significant distress as a result of a relationship break-up and took an overdose because of her distress. The bond with the psychiatrist fails when SP1 believes the psychiatrist finds her to be overreacting and not requiring hospitalization despite being suicidal.

“[I feel] like I was standing in front of a cold wall analyzing me,”… I’m overplaying my heartbreak. [A]t one point she says, ‘ah, you’ve got a broken heart, you know, that’s basically it, you’ve got a big broken heart.’ So I don’t think she trusts me when I say I need help and all that. I [didn’t] trust her either” (SP1, P3).

SP2 has diabetes and is experiencing a lot of anxiety concerning her blood sugar fluctuations, so she has been referred to a psychiatrist for an assessment. For her, the bond fails when a psychiatrist does not take her fears seriously or quickly pathologizes them without understanding why she feels as she does.

“He sees the theories that apply, or he sees the overlap between me and what he has seen in his books or with other patients…[I] didn’t feel listened to like a person, the interaction felt very procedural: a checkbox… I think it’s important… for the patient to feel like an individual and not a case” (SP2, P7).

SP3 believes he is being conspired against at work. For him, the bond fails when a psychiatrist doesn’t try to understand his beliefs but instead jumps to confronting him about his psychotic symptoms.

“Sometimes I wonder… is his empathy sincere or not? Because of that confrontation, …in that judgment, I also sometimes felt a kind of distance from me, like… he wasn’t completely empathic” (SP3, P16).

Thus, bond formation —and the TA by extension — are facilitated when SPs believe they are respected by psychiatrists through different means and at different phases of the diagnostic encounter. Our analysis suggests that psychiatrists have the additional task of accurately discerning how respect and disrespect are conveyed for a particular individual and adapting their styles accordingly.

## Discussion

This study contributes to ongoing debate about the relational validity of simulated clinical encounters in psychiatric and psychotherapeutic education. Our results support the view that bond formation can occur between an SP and a psychiatrist in a full-length psychiatric assessment from the SP’s perspective. Although the three SPs played patients with different clinical problems and met with different psychiatrists, the factors that favoured bond formation were convergent across their encounters. Specifically, psychiatrists’ respect, both in trying to understand the SPs problems and in working towards potential solutions was an essential ingredient in facilitating SPs perceptions of bond formation. While the importance of respect was present across SPs and encounters, SPs had different preferences as to how it should best be conveyed and different views as to what counts as disrespect. This meant that in addition to conveying respect, psychiatrists had the additional task of accurately discerning how respect (or disrespect) is manifest for a given SP and adapting their style accordingly. Some psychiatrists were able to convey respect in the way preferred by the SP, and succeeded in fostering a strong bond. Importantly, what SPs value in bond formation and what went wrong when bond formation did not succeed, are the same things that facilitate or compromise bond formation in practice between clinicians and real patients [25]. Therefore, our analysis offers some evidence that from the SP point of view, simulation is not a barrier to bond formation.

The design of our study is unique in certain ways which is both a strength and may serve as a useful approach for future research in the field. While most of the research involving SPs has been conducted using shorter encounters such as OSCE stations, our interviews more closely replicated usual practice with standard evaluations lasting 45–60 min in real life settings. Asking the SPs to review the full videorecordings for each of their role plays and their WAI ratings as elicitation techniques strengthened our data by facilitating recall of tacit and contextual details. Finally, our analysis highlights the SP perspective in simulations where building a therapeutic alliance is an important aspect of what is being taught or assessed. Because patient perception of the therapeutic alliance is an important predictor of outcome, SP perspectives can offer valuable formative feedback to learners.

### Limitations

Our findings must be considered in the light of certain limitations. First, the lack of psychiatrist ratings did not permit us to compare the SP with psychiatrist perspectives in order to draw conclusions about alliance formation from both parties in the relationship. In particular, the absence of psychiatrist ratings did not allow conclusions about whether simulation affected alliance formation and potentially, clinical performance, from the clinician’s perspective which has been an important preoccupation in the literature. Second, the presence of an observer filming the assessment throughout the interaction may have contributed to the ease (or unease) of bond formation. While our data do not suggest that this affected our SPs, it may have affected the psychiatrist-participants and their behaviour which in turn may have affected the bond from the SP perspective. Finally, while one of our SPs also suffered from a condition they portrayed, ideally, the scenarios would all be designed in collaboration with people with lived experience to ensure authenticity.

## Conclusions

This study contributes to the literature on the participation of SPs in psychiatric and psychotherapeutic education, particularly to teach and assess relational competencies. Our results suggest that SPs can form a bond with psychiatrists in full-length diagnostic assessments. Bond formation was facilitated when psychiatrists conveyed respect, in the preferred way, towards the SP. Respect was essential both when psychiatrists attempted to understand the SP’s problem and in their approaches to finding solutions. These factors are the same factors that patients identify as facilitating bond formation in real practice. These results provide preliminary evidence that from the SP perspective, the simulation itself was not a barrier to bond formation.

Future research in this area would compare SP and clinician ratings in the same encounter in order to determine whether bond formation had occurred from both perspectives, the extent to which there is agreement on the factors that facilitate bond formation, and therefore the relational competencies that should be taught and assessed. In addition, research that obtains TA ratings of individual clinicians by several SPs, and TA ratings of individual SPs by several clinicians, would help to shed light on the extent to which bond formation is the result of the specifics of the encounter, including the simulation, versus clinicians’ or SPs' relational strengths and deficits.

## Supplementary Information

Supplementary Material 1.

## Data Availability

The data that support the findings of this study are available upon request.
