# Supplementary material for: “Meeting a human, not a medical condition”: a qualitative study of simulated patients’ perceptions of forming a therapeutic alliance with psychiatrists
Source: Adv Simul (Lond). 2026 May 23;11:52. doi: 10.1186/s41077-026-00446-7 (PMC13393675; doi:10.1186/s41077-026-00446-7)
Supplement: Supplementary file 1 [file 41077_2026_446_MOESM1_ESM.odt]

APPENDIX A - SP SCENARIOS

Situation: Emergency Room Consultation

Reason for Referral: overdose

This scenario is designed to mobilize the feelings of the clinician so that we can understand how (or if) physicians use their own feelings when trying to make a diagnosis.

Instructions to the actor: this patient is easily irritated and difficult to pin down. She easily evades the question or changes the subject.

Ms C

30 year old woman, working as a salesperson for a large furniture company for the last three years. Involved in a relationship for the past 8 months with a 29 year old man who is a musician. She has been living in his apartment after two months of being together (they have been living together for 6 months).  She is in the Emergency Room following an overdose of acetaminophen Her parents live in another town. She was brought to the ER by a neighbour.  She has a sister aged 25 who is a university student.

She says that she has been feeling very down lately because she believes her relationship is over.  Tonight she was alone in her apartment and had drunk several glasses of wine. She was ruminating about her boyfriend leaving her and couldn’t take it anymore. She decided to overdose thinking that she would be better off killing herself than having to live without him.  By chance her neighbour had come to the door and she told her what she had done. The neighbour insisted that they come to ER.

She met her boyfriend at a party and was thrilled when he noticed her. She thinks he is like ‘a movie star’ and attracts attention from everyone. He plays the guitar in a band that is gaining more attention. There are always sizable crowds when they play and lots of women crowd the stage.  She has felt very special during these past months with him although occasionally feels jealous, especially when he is talking with fans after the show.  He gets angry when she accuses him of flirting with the fans and says that he has to be friendly, it is part of the business. 

Over the past two months he has been growing increasingly distant.  He has been going out to rehearsals with the band and coming home later. Sometimes she waits up for him and often on these occasions she notes that he smells of alcohol. Sometimes he gets home after she goes to bed. She is not sure if she believes him when he says where he is going because once she called one of the other bandmates during a rehearsal but there was no rehearsal that night. When her boyfriend found out she called they had a huge fight. He told her to ‘get off his back’ and that sometimes he ‘needed his space.’  On another occasion, she followed him out of the apartment and saw that he entered a building she didn’t know. She confronted him when he returned and they had another big fight.  She says that they argue frequently but is not particularly bothered by this. She denies that they are physically aggressive with each other although says they might throw things if the fight is really escalating. Afterwards, they often have ‘great makeup sex.’

Lately, she has been worrying that he is going to leave her. He has said that he is not sure if he is  ‘into her’ anymore.  She feels very hurt by this. She finds herself preoccupied by worries about losing him which she finds unbearable. Although she has had several relationships before this one, most were short-term, 1-2 months, and she has never felt as seriously about someone as she has about this man.  She has fantasized about getting married and having a family. She can imagine him being a famous rock star and she would be traveling along with him with their children.

When he goes out without her, she will often stay at home and have a few glasses of wine and listen to music.  When asked about her alcohol consumption, she becomes annoyed and says her consumption is completely normal.  Prior to this relationship it was her usual pattern to go out every weekend and drink to the point of intoxication on Friday and Saturday nights. She has never had any trouble with the law and has never gone to work intoxicated or hung over.  Once she had a bad fall from her bike when she was riding home drunk. She will occasionally use street drugs with her friends at parties but nothing regularly.

She often feels alone even though she has friends. She describes her friends as ‘party people’ who don’t really know her true self. She is afraid to show them ‘what I am really like.’  They are people she knew in college but are part of a larger circle of friends. The three people she was closest to have settled into long-term relationships and two have young children. She sees them rarely. She tends to see her current friends on weekends when they are going out. Since she has been with her boyfriend, she has been calling them less and less. 

She says she has been feeling increasingly depressed. She is also angry. She has been sleeping erratically, sometimes oversleeping, sometimes not sleeping because she is up worrying that her boyfriend is with other women. She doesn’t think she could ever find a relationship as worthwhile as this one again.  She is able to do her job as usual although is less interested in it now.  She had been quite successful at sales, regularly exceeding targets and having been top salesperson of the year last year. 

She says that she is feeling less like ending her life now that she is in the ER. She tells you that she wants to stay in hospital so that she can recover from this relationship and ‘get the help that I need.’ If she leaves, she is sure that she will overdose again.  Life wouldn’t be worth living without her boyfriend.

Past History

There is no past medical history and she uses no medications.

She saw a psychiatrist as a teenager when she suffered from anorexia nervosa. She was not admitted to hospital during that period and thought the psychiatrist was ‘a bit of a jerk.’ But she thought that the social worker she saw was very nice. She was prescribed some kind of ‘happy pills’ but took them erratically. The problem passed after two years although when she is upset, she might ‘forget to eat’ because she is so distressed.

She has not come to the psychiatric ER before. She admits to have overdosed before on Tylenol on two prior occasions but she didn’t ‘feel like’ coming to ER.  She does not endorse any other self-harming behaviours.

She does not know if there is any mental illness in her family but notes that her mother is very difficult and demanding.  Apparently her maternal aunt has been to see many psychiatrists. The patient doesn’t know what is wrong with her but says that everyone agrees she is often in distress.

Mother is 60 and Father is 58.  Both are in good health as far as she knows.  Both parents have one sibling and neither are close to the family.  Grandparents are all deceased, but she remembers both grandmothers well.  They were not that close as they did not live nearby, but says they were ‘nice.’ 

She smokes one half a package of cigarettes per day.

She describes a ‘strange’ (her words) childhood but has difficulty elaborating on what she means. Her father was quiet and distant, her mother was often angry about her father’s distance or about her daughters’ various ‘failings.’  Her sister was quiet like her father and did well in school. Her mother complained that she didn’t socialize enough – how would she find a husband? The patient was popular and less successful academically than her sister. Her mother was disappointed in her grades and told her she needed to pay less attention to boys and more to her studies. She sees her parents a few times per year for holiday gatherings ‘which is enough, she can be a total bitch.’  She is not close to her sister but thinks well of her.

After she finished school, she went on to attend college where she studied business. She has had several jobs although they never lasted longer than a year until this one. She is vague when asked about why she has such an erratic employment history. She tells you she likes this job better than the others, ‘there are a lot of cute guys in this business!’

Mental Status:

Young woman, looks her stated age. Attractive, constant eye contact, dressed fashionably.  Occasionally dramatic in her speech and gestures but otherwise it is normal in rate and rhythm.  She describes herself as ‘very depressed.’  Her affect is irritable. But varies somewhat according to the content of the interview. Her thoughts are goal-directed but she sometimes has difficulty elaborating. She is preoccupied by worries about the ending of the relationship.  She appears to be of normal intelligence. Her insight into her situation is moderate.  Her judgment during the appointment is appropriate.

Situation 2:  Outpatient Psychiatry Consultation

Reason for Referral:  excessive worry about developing a medical condition

This scenario is designed to have the participant weigh the patient’s self-report of symptoms and of her hypothesis against the lab data and his/her knowledge of the referring doctor.

Instructions to the actor: this patient is earnest, sincere, and convincing.

Ms B

28 year old woman, married, one child (15 month old daughter)
Works in a full-time in a high-end retail store selling women’s clothes
Attended high school and a two year college course in fashion
Would like to move up in the company, perhaps as a buyer, but is content in her current job.

She is referred to see a psychiatrist by her endocrinologist because of ‘anxiety.’

Her endocrinologist is well-known to the psychiatrist, she sends appropriate referrals regularly, and is always receptive to your feedback.

Ms B has had type 1 Diabetes Mellitus since the age of 21. She is on a four times daily injection schedule of insulin which she takes regularly. She was also diagnosed with hypothyroidism which started shortly after the birth of her daughter.  It was discovered incidentally on a routine endocrinology appointment. She remembers being tired, but because she was just a few months post-partum, she thought it was due to that.  Her doctor prescribed thyroxine and she has been taking it regularly.

She believes there is something wrong and doesn’t think she is being taken seriously by her doctor. She has found that since she had her baby, that her glycemic levels have become much more unstable. She can eat something one day and have normal sugars and the next day, she can eat the same thing and find that it leads to hyperglycemia.  She says her blood sugars can swing between 20 and 6 in just a few hours. This is now happening several times per week. She has restricted her diet to one that is gluten-free to try to better control her blood sugars as she thinks there is a pattern with gluten consumption and hyperglycemia.  She has also seen a naturopath who told her she had high levels of yeast and she wonders whether adopting a yeast-free diet would work. She plans to try it. 

She has seen her endocrinologist on three occasions about this problem. The first time, she adjusted her insulin dosing. The second time, she did the same but also referred the patient to see a dietician to review her nutritional intake. On the third appointment, the endocrinologist implied that the patient must be exaggerating about the swings in her sugars. She asked the patient if she would see a psychiatrist because she was worrying too much and maybe that was leading her to  ‘play around’ with her diet too much.

With the passage of time, the patient is becoming increasingly concerned about what is causing the instability in her sugars.  She wonders if maybe she has some kind of cancer that is causing the problem. She remembers a friend in college who hadn’t been feeling well for several weeks and went to see two different doctors in walk-in clinics who told him they couldn’t find anything wrong. Finally he went back again and he found out he had some kind of cancer.  She remembers that he had treatment for several months, seemed to be getting better but then suddenly he got worse, went to the hospital and died there.  Even though this episode was very upsetting, she hasn’t been overly preoccupied with it over the last year.

Ms B does worry a great deal about this issue although she does not tend to worry about other things. She says her family life is happy and she and her husband are financially comfortable. Her husband is a junior manager in a large company. ‘I wouldn’t say no to winning the lottery, but we’re ok.’ Her father died a year ago from colon cancer but her mother is well. She lives near Ms B’s brother. Sometimes Ms B has trouble sleeping because she is worrying about her blood sugars going low. This did happen once but she woke up and was able to take the steps to bring it back up. She says that she is often irritable but attributes this to her blood sugars. ‘Do you know what it is like to be at 25?’ She is often tired but attributes this also to changes in blood sugars and having to work while also looking after her daughter.  She can often be constipated. She often feels cold but says this has always been the case.  She is having trouble losing her pregnancy weight.

She says she feels sad when she thinks about her father. She does not report any suicidal thinking. She is able to do her job and continues to enjoy it and spending time with her family. She does not report any symptoms consistent with psychosis or mania. She feels very annoyed with her endocrinologist.

Past History
She saw a psychologist at age 16 when her parents separated. She was very upset by the separation. The psychologist helped her ‘to get through it.’ She remained close to both parents even after the separation.

She does not smoke cigarettes, or use street drugs. She does have a glass of wine most evenings at home after her daughter goes to bed.

Mother is 58. Father died at age 59.  Brother is 30.  Mother is in good health. Brother had asthma as a child and was frequently in and out of emergency rooms and hospital. It is much better now and well-controlled with treatment.

There is no psychiatric history in the immediate family but there is a maternal aunt with epilepsy.

Ms B has no other medical problems and takes no medication apart from insulin and thyroid hormone.

Ms B describes her childhood as good.  Her parents were often in conflict which she recalls from an early age.  There was no violence in the home. She was not very interested in school and performed at an average level. She had several friends throughout her schooling.   Her brother was a strong student who did well and went on to university.   He now works as an engineer. He is single.

Ms B was more distraught by the separation than her brother, even though she was not surprised when her parents announced the news.  Her brother did not react much.  They have always had a friendly relationship but do not confide in each other.

Diagnosis of diabetes at age 21 following a 2-3 week history of classic symptoms: thirst, polyuria, weight loss, feeling unwell.  Her mother accompanied her to the ER where the diagnosis was suspected. She was admitted for a few days during which time her blood glucose was brought under control and she was taught the basics of diabetes self-management.  She says she has always followed the treatment and sees her endocrinologist 2-3 times per year. She has only had the current endocrinologist since the year before pregnancy when her former endocrinologist moved away.  They get along but their relationship is quite practical and technical. Patient found the demands for very tight control during pregnancy very stressful and said that she tried to talk to her endocrinologist about this. The specialist responded by saying that ‘everyone finds it hard but you’re doing great.’

Mental Status
Non-contributory

Lab Results

TSH -  5.0
T3 – normal
T4 – normal

HbA1C – 0.68

Situation 3: Outpatient Psychiatry Consultation 

Reason for Referral: Change in behaviour, more withdrawn

This scenario is designed to have the participant to weigh the testimony of the mother against the self-report of the patient.

Instructions to the actor: The patient should be passive and neutral.

Mr A
21 year old man
Lives with his parents.
One sibling, a sister who is 30, married and lives elsewhere
Attended University for two years (English) but decided to take some time off to think about what he really wanted to study and do in his career
Working at Home Depot in the paint department. He has always had good aesthetic sense and enjoys helping people plan the colours for their living space
 
Referred to see a psychiatrist by a GP at a walk-in clinic at his mother’s request. Both mother and son had attended the appointment. The young man’s mother told the GP that she thought there might be something ‘mentally wrong’ with her son because he seemed paranoid in the last several months. Apparently, he had been complaining that some of the co-workers and supervisors at Home Depot didn’t like him, were treating him badly, and had even threatened him.  However, when she stopped at the store one day when he was working, she talked to a few of them and they seemed like ‘nice young people.’  She has also noticed that he has not been socializing very much in the last several months and that he doesn’t have a girlfriend. The GP agreed to provide the referral as the son did not resist. 

The patient explains that his mother and the GP think that he is paranoid but he is not. They don’t work at the Home Depot, how would they know? He asks if he can explain the situation?  

This is what he describes:
He has been working at Home Depot for just over a year in the afternoons and evenings. For the first several months, things were fine.  At that point a new supervisor was hired. This man spent a lot of time checking up on people, their hours, overseeing their work.  He was not the only one targeted. Then about 4 months ago, the supervisor made a sexual advance at him. In retrospect, the patient thinks that perhaps the supervisor had been trying to give him hints all along that he was interested because he seemed to spend more time with him than other staff and would occasionally ask questions ‘what are you doing on the weekend?’ or did he ‘have plans with his girlfriend?’  This latter question prompted the patient to reply that he didn’t have a girlfriend.  

When the sexual advance (supervisor placed his hand on the patient’s knee during lunch)  was made, the patient rebuffed it and thereafter, tried to stay away from the supervisor. It seemed from that time on, that the supervisor was getting together with other staff and gossiping about him.  The supervisor would tell others that he (the patient) was gay but ‘in denial.’ Apparently, he also told certain co-workers that the patient had told the supervisor these co-workers were gay.  Some of these details were told to him by co-workers, others he overheard when he entered the break room.  Gradually the other staff started interacting with him less and less, and wouldn’t help him with various tasks. The patient saw a stranger in a vehicle parked outside his house on at least three occasions whom he thinks was hired by co-workers to either scare him or collect information about him. He says ‘some of those guys (co-workers) are real thugs and have ties to the mafia.’  The patient requested shifts that were at different times than these co-workers which was granted.   He noted that the new co-workers seemed friendly at first, but then after a short period became cold and would not help him just like the others.

Meanwhile, the supervisor has complained about him on the grounds that he was not working efficiently and has tried to get him fired.  The complaint was not sufficiently substantiated so there were no consequences.  The patient believes that it is just a matter of time before the supervisor ‘plants drugs on me or something like that’ in order to get him fired. 

In response to his mother’s concerns, the patient explains that he is not socializing because his friends were people from work but now they are against him. ‘Of course I don’t want to be hanging out with them.’ He says that he does not have a girlfriend because he hasn’t met anyone yet.  He jokes ‘does that make me gay?’

Past History
There is no past medical history and he uses no medications.
He has never seen a psychiatrist before. 
He believes his mother suffered a depressive episode after his birth and thinks it was serious because she and his father have referred to her hospitalization.  He doesn’t know if she suffered from depression after the birth of his sister.
His father has type 2 diabetes. His mother and sister are medically well.

Mother is 51 and Father is 52.  Father works as a manager in a construction company, mother has always been at home. Mother was one of three children and father was an only child. Maternal grandparents are both dead, grandmother in the last year. Mother was and always had been, very involved in her care. Px thinks that grandmother may have also had a psychiatric history as his mother has alluded to her having to go to the hospital sometimes. Father’s parents are both alive and spend 7-8 months per year in Florida so he doesn’t see them much but when they are back, they visit once per week.

He does not smoke. He drinks alcohol when he is out with friends and might drink to the point of intoxication in these situations but never more than once per month. He has smoked a joint with some of his co-workers after they are finished at work but this is quite occasional, once every month or two.  He has no legal history. 

The patient describes a ‘normal’ childhood. His parents were ‘regular parents.’  They were loving, his mother was more outgoing than his father who was and remains quite reserved. They were always pleased by any success he had. He likes his sister but they were not close due the age difference. She had left home when he was still in primary school. The patient played hockey for several years as a child but then realized he didn’t really like it that much and gave it up at age 13. He was an average student but excelled in creative writing and literature which is why he decided to study this at University.  Near the end of high school he became involved in the drama club which he liked although he found putting on the annual production quite stressful.  Some of the other kids were jealous that he was cast in a big part even though they had been in the club longer than he had. He realized that he found the technical aspects of theatre more interesting than acting. He liked planning and designing the set and props the most.  He had a few friends in childhood but after he stopped playing hockey, he did not see them so often.  He had a few casual friends in high school but was often busy with homework, writing and reading.  He dated one woman in university whom he met in class. They broke up after a few months because ‘there was no chemistry.’ He thinks that she was more interested in him than he was in her. After two years of university, he was no longer sure that he wanted to continue in the degree. He loved literature but he didn’t know if he loved studying literature and he had no idea what kind of a job he wanted after university.  He decided to take some time to think about it as he didn’t want to end up like ‘those people who stay in university because their parents want them to.’

Mental Status:
Young man, looks a bit older than his stated age. Mildly unkempt but hygiene appears appropriate. He makes only occasional eye contact. There is no psychomotor agitation or retardation. His speech normal in rate and rhythm but he occasionally becomes loud and animated when he is describing recent events at work.  He describes his mood as ‘nervous’ to be telling all of this to a psychiatrist (‘could I get in trouble at work?’) but his affect is neutral and with little range. He denies any hallucinations. These thoughts are clear and goal-directed. He is preoccupied by the people at work, their behaviour, and possible repercussions to his safety. He appears to be of normal intelligence. His judgment during the appointment is appropriate.

In the scenario, the psychiatrist has received the following letter from the patient’s mother, but the patient himself has not read it.

Mother has written a letter to the psychiatrist which says the following:

Dear Doctor,

 
I hope that it is appropriate that I am writing this letter. I couldn’t be at the appointment today but I wanted to share my perspective on how my son has been doing over the last several months.  

He has been acting very strangely, keeping to himself and not going out except to work. He spends most of his time in his room when at home. He used to love his job but now when he talks about it, he sounds ‘paranoid’ if I can use that word, saying that his boss and co-workers are out to get him. I am sure he is going to tell you all about this.  I don’t know these people of course, but the few times I have interacted with them, they all seemed so friendly and helpful. I have no idea what he is talking about. I also worry that he has no girlfriend. He is 21 years old and doesn’t ever have girls calling the house or coming around. He did date someone a few years ago but that was only for a short time. I met her once and really liked her.

I am concerned that there is something seriously wrong. I should tell you that I had depression twice before and once I had paranoid ideas myself. The first time was after my son was born.  I had ideas that they would take him away from me so I should leave town with the baby and never come back.  I was admitted to hospital for a few days for treatment. I had another episode about 10 years later but this time no paranoia.

My son has never been extremely outgoing, but this is much worse than before.

I hope you find this information helpful. Please call me if you need more information.

Thank you,

Mrs X

APPENDIX B - DRS INTERVIEW GUIDE 

A. 	Questions for the researchers when reviewing the videos

1.	about specific moments in the video

a.  	What is happening here?

b. 	How would you describe:

                            	i. 	the psychiatrist? (emotions, active/passive, (non)directive, verbal/nonverbal language)

                           	ii. 	the patient? (emotions, verbal/nonverbal language)

                          	iii. 	their interaction or connection?

c.  	How might a psychiatrist use information from the interaction with the patient when formulating a diagnosis and/or treatment plan?

d. 	What other types of information would be useful when forming a diagnosis or treatment plan?

2.	after reviewing the entire video

a.  	Overall how would you describe the psychiatrist during the visit with the patient?

b. 	How would you describe the patient?

c.  	How would you describe their interaction or connection?

d. 	What sources of information might be useful when forming a diagnosis or treatment plan for this patient?

 

B. 	Questions to ask the psychiatrist-participants

1.	about specific moments in the video; the ones selected in Section A1 above 

a.  	What is happening here?

b. 	How would you describe:

                            	i. 	yourself at this moment? (feelings, thoughts, behaviours (level of passivity/activity, verbal, nonverbal)

                           	ii. 	the patient at this moment? (emotions, verbal/nonverbal language)

                          	iii. 	your interaction or connection with the patient?

 

2. 	about the taped session, after reviewing the entire video

a.  	Overall how would you describe yourself during the visit with the patient?

b. 	How would you describe the patient?

c.  	How would you describe your interaction or connection?

d. 	What diagnosis or differential diagnosis did you arrive at?

e.  	 What sources of information make you favour that diagnosis (or favour one versus another if there is a differential) ? [interaction with patient, others]

f.   	How did you sort out conflicting information such as your clinical impressions from your interaction with the patient and testimony (from family, lab results)?

 

3. 	about [his/her] clinical theoretical stance or framework

a.  	What kind of theoretical stance do you adopt when you see a patient for the first time?

b. 	Is this the approach you were trained in, or have you developed it since then?

c.  	How would you describe the clinical framework(s) that shaped your interactions with this patient?

d. 	How does this stance influence your diagnostic reasoning?

 

4. 	about  other patients or cases [she/he] has treated

a.  	Think back to other patients you have treated who remind you of this one. In what ways were they similar to/different from this patient?

b. 	Has working with [him/her/them] influenced your diagnosis with this patient? If so, how?

c.  	Are there certain types of patients that you find easy to work with? If so, why?

d. 	Are there certain types of patients that you have difficulty working with? If so, why?

e.  	Can you think of a case in which you had difficulty making a diagnosis? Can you explain why it was difficult?

f.   	 

 

5. 	about clinical reasoning in psychiatry

a.  	What makes a good psychiatrist?

b. 	What roles does intuition play when making a diagnosis?

c.  	What about empathy; where does that fit in? 

d. 	How do you evaluate conflicting information, such as knowledge you gain from directly interacting with a patient and testimony from a family member?  What about conflicting reports of symptoms by the patient and lab results?

6. 	wrap-up

a.  	What advice would you give us about how to train the next generation of psychiatrists? 

b. 	Any last thoughts?

APPENDIX C - WAI CLIENT VERSION

The full scale is available here: https://wai.profhorvath.com/downloads

The bond subscale refers to the following questions of the WAI client version: 1, 5, 8, 17, 19, 20, 21, 23, 26, 28, 29, 36

All questions are rated on a 7 point Likert scale with the following anchors:

1                2              3                       4	              5               6                        7

Never       Rarely     Occasionally     Sometimes         Often        VeryOften	      Always

1. I feel uncomfortable with _________.

                         		                 

5. _________ and I understand each other.

8. I believe _____________  likes me.

17. I believe _______________ is genuinely concerned for my welfare.

19.  _______________ and I respect each other.  

20. I feel that _______________ is not totally honest about his/her feelings toward me.

21. I am confident in _______________ 's ability to help me.

23. I feel that _______________ appreciates me.

26. _______________ and I trust one another.

28. My relationship with _______________ is very important to me.

29. I have the feeling that if I say or do the wrong things, __________will stop working with me.

36. I feel _____________cares about me even when I do things that he/she does not approve of.

APPENDIX D - SP INTERVIEW GUIDE

1. Can you explain why you gave this rating (for any question rated 1, 2, 6, 7)?

 

2. At the end of all the videos: How did your experience differ from one interview to the other?

 

 

Probes:

 

“I don’t know”

 

I see that you rated the interviews differently.

Specifically, this question.

 

Why is that?

Why do you think that is?

Can you think of an explanation for this?

 

Do you think it was related to the fact that you were an actor?

 

 

“They were all the same”

 

Psychiatrists approached you differently, why do you think that is?

How was the interaction between you two different?

How did this compare to your other experiences as a patient?

You acted differently. Why?
